# Evaluation of Thromboelastography 6s prognostication of fibrinogen supplementation in pediatric cardiac surgery

**DOI:** 10.1111/aas.14144

**Published:** 2022-09-11

**Authors:** Rasmus Bo Lindhardt, Jonas Rønne Kronborg, Michael Wanscher, Lars Willy Andersen, Jakob Gjedsted, Hanne Berg Ravn

**Affiliations:** ^1^ Department of Cardiothoracic Anesthesiology, Rigshospitalet Copenhagen University Hospital Copenhagen Denmark; ^2^ Institute of Clinical Medicine, Health Faculty University of Copenhagen Copenhagen Denmark; ^3^ Institute of Clinical Medicine University of Southern Denmark Odense Denmark; ^4^ Department of Anesthesiology and Intensive Care Odense University Hospital Odense Denmark

**Keywords:** cardiopulmonary bypass, cryoprecipitate, pediatric cardiac surgery, point‐of‐care test, thromboelastography, transfusion

## Abstract

**Background:**

Implementation of point‐of‐care tests is recommended to provide tailored substitution during cardiac surgery. The measurement and substitution of fibrinogen have gained particular interest since it is the first coagulation factor to become depleted during cardiac surgery. However, the prognostic ability of thromboelastography (TEG) 6s has not been evaluated in pediatric patients.

The aim of the present study was to describe patient characteristics of infants receiving fibrinogen substitution during cardiac surgery and evaluate the prognostic ability of TEG6s after weaning off cardiopulmonary bypass (CPB).

**Methods:**

Infants undergoing congenital cardiac surgery with CPB were retrospectively included (*n* = 279) between January 2017 to July 2019. Patient and perioperative data were collected on the day of surgery until 6:00 AM the next morning. Hemostatic capacity was assessed with TEG6s.

The efficacy of TEG‐functional fibrinogen‐maximal amplitude (TEG‐FF‐MA) measurements for the prediction of intraoperative bleeding, and thereby cryoprecipitate need, was evaluated by a sensitivity and specificity analysis.

**Results:**

Among 174 children with TEG‐FF‐MA data, 147 (84%) received cryoprecipitate intraoperatively. Cryoprecipitate administration was associated with younger age 66 (10–132) versus 98 (45–204) days (*p* = .044), higher RACHS‐1 classification, and intraoperative bleeding 21 (11–47) versus 5 (3–13) ml/kg (*p* < .001, mean difference 29 ml/kg [CI: 8–50]). Median TEG‐FF‐MA values were lower in transfused children 7.6 (5.3–11.0) versus 10.5 (7.3–13.4) mm (*p* = .004, mean difference − 2.4 mm [CI: −4.1 to − 0.73]).

The volume of cryoprecipitate was associated with bypass time, TEG‐FF‐MA values, and in particular intraoperative bleeding volumes.

A TEG‐FF‐MA threshold of 10.0 mm, resulted in sensitivity: 74%, specificity: 56%, positive predictive value: 80%, and a negative predictive value of 47% for the prediction of intraoperative bleeding (>10 ml/kg) and consequently a need of cryoprecipitate transfusion.

**Conclusion:**

Fibrinogen substitution in infants was associated with younger age and higher RACHS‐1 category.

The prognostic value of TEG6s was evaluated, and cryoprecipitate transfusion was related to TEG‐FF‐MA values, but also CPB‐time, surgical complexity, and in particular excessive intraoperative bleeding. A clear‐cut threshold for TEG‐FF‐MA is difficult to establish in infants undertaken congenital heart surgery.

**Editorial Comment**

This study identified several risk factors for cryoprecipitate administration and the transfused volume in pediatric cardiac surgery. The cryoprecipitate transfusion was related to thromboelastography‐6s (TEG‐6s) functional fibrinogen‐maximal amplitude (FF‐MA) values, bypass time, surgical complexity, and in particular excessive intraoperative bleeding.

## 1 INTRODUCTION

Congenital heart surgery with cardiopulmonary bypass (CPB) is associated with significant morbidity and mortality. A great component of this risk stems from perioperative coagulopathy resulting in increased bleeding.^1^ An increased bleeding risk relates to multiple causes in children, including hemodilution. This hemodilution is relatively greater in infants than adults, since the ratio of the CPB priming volume to the child's blood volume is increased.^2^

Other risk factors for increased perioperative bleeding in pediatric patients include an immature coagulation system, as well as hypothermia, and extensive, complex surgery leading to increased CPB duration, too.^3^, ^4^

Due to surgery and perioperative coagulopathy, the vast majority of infants require transfusion of blood products during and after surgery. Consequently, transfusion management has become an increasingly bigger part of the perioperative optimization during pediatric congenital heart surgery. Therefore, the majority of pediatric cardiac centers now apply point‐of‐care tests like rotational thromboelastometry (ROTEM) or thromboelastography (TEG) 6s when weaning off CPB to obtain early detection of coagulopathies, and improve patient outcomes.^5^ Point‐of‐care test‐guided transfusion management during or immediately after CPB has in previous studies been shown to reduce not only the volume of blood products, but also the proportion of patients receiving transfusions.^6^, ^7^ Despite these advantages, there is still debate regarding the threshold for individual viscoelastic parameters to give targeted blood products.

Fibrinogen is the first coagulation factor to become depleted in cardiac surgery,^8^ and for this reason, major interest has been devoted in adult cardiac surgery to establish an algorithm, where point‐of‐care tests or measurement of the fibrinogen concentration can guide the clinician with the identification of patients, where fibrinogen should be substituted prophylactically. So far, this endeavor has been in vain—and administration of fibrinogen would in many situations lead to unnecessary substitution.^9^

In children, low fibrinogen levels immediately after CPB have been associated with postoperative bleeding in a single observational study.^10^ Cut‐off levels have been published for ROTEM, but never for the newer TEG6s test in a pediatric population. Test performance is to some degree dependent on patient selection and since fibrinogen substitution occurs more frequently in younger patients, we chose exclusively to include infants in the study, to evaluate the performance of the newest point‐of‐care test: the TEG6s. The present study aimed to describe patient characteristics of infants receiving fibrinogen during cardiac surgery, and additionally to evaluate the prognostic ability of TEG6s to identify the need for fibrinogen substitution.

## 2 METHODS

A retrospective, single‐center, observational study with children undergoing pediatric congenital heart surgery at the Heart Center, Rigshospitalet, Denmark.

### 2.1 Patient inclusion and management

Children <1 year of age undergoing elective cardiac surgery with the use of CPB from January 2017 until the end of July 2019, for one or more surgical procedures due to congenital heart defects.

All children had a preoperative screening test of standard laboratory coagulation parameters performed, including platelet count, aPTT, and INR. Laboratory results were interpreted according to age‐related reference intervals and treated in case of bleeding. None of the infants received anticoagulant or antiplatelet treatment prior to surgery, and none suffered from hereditary bleeding conditions.

Children were undertaken anesthesia by inhalation of sevoflurane followed by intravenous fentanyl 5–10 mcg/kg, midazolam 0.05–0.1 mg/kg, and rocuronium 0.5–1.3 mg/kg before intubation with an adequately sized nasal endotracheal tube. Every infant was cannulated with an arterial and a central venous line. They all had a urinary catheter with a thermistor to measure core temperature and a peripheral thermistor for skin temperature measurement. Children were kept at a target core temperature of 34°C, the only exception being aortic arch surgery, where the core temperature was lowered to a target of 25°C.

Cardiopulmonary bypass was conducted in accordance with the weight of the child.

Children weighing <7 kg, underwent cardiopulmonary bypass utilizing a Capiox® FX05 oxygenator (Terumo, Ann Arbor, MI, USA) with a non‐pulsatile flow of 2.8–3.2 L/m^2^/min and 3/16‐inch inlet tubing. Children weighing >7 kg underwent cardiopulmonary bypass utilizing an Affinity Pixie™ oxygenator (Medtronic, Minneapolis, MN, USA) with a nonpulsatile flow of 2.6–2.8 L/m^2^/min and ¼‐inch inlet tubing. The CPB prime consisted of 250 ml SAG‐M, 100 ml human albumin 20%, 25 ml sodium bicarbonate 8.4%, 70 ml Plasma‐Lyte, 3 ml calcium chloride, and 0.2 ml heparin 5000 IU/ml in children <7 kg. An additional 100 ml of plasma‐lyte was added to the prime in children >7 kg. All children were heparinized to a blood concentration of 3.5 mg/kg as measured by HMS Hepcon® point‐of‐care test (Medtronic, Minneapolis, MN, USA) or an activated clotting time > 480 s. Protamine was later given according to HMS Hepcon results. All children underwent antegrade cardioplegia with a 4:1 blood to cardioplegia solution ratio in a 25 ml/kg loading dose with a following 10 ml/kg maintenance dose every 20 min. Lastly, hematocrit was kept between 28% and 32% in all children during CPB. When weaning from CPB, children underwent modified ultrafiltration to a hematocrit of 40%–42% in children <7 kg, and a goal of 35%–40% in children >7 kg.

Hemostatic capacity was evaluated using TEG measured with TEG®6s (Haemonetics Corporation, Boston, MA, USA) and platelet aggregation with Multiplate® (Roche Diagnostics, Rotkreuz, Switzerland), respectively. Both TEG6s and multiplate tests were measured at the end of cardiopulmonary bypass after rewarming to at least 36**°**C. The samples were analyzed at 37**°**C in the presence and absence of heparinase. Only heparinized measurements are reported. Children were transfused if there was ongoing bleeding and TEG‐FF‐MA < 10 mm (cryoprecipitate), and platelets if TEG‐MA < 45 mm AND TEG‐FF‐MA > 10 mm or if multiplate aggregation responses (ADP, ASPI, and TRAP) were below 20% for all agonists. During major bleeding, volume substitution is recommended with a ratio of red blood cells: cryoprecipitate: platelet pools of 2:2:1. Once bleeding got under control, the institutional recommendation is to consider reassessment by performing a new TEG6s and/or Multiplate measurement.

### 2.2 Data collection

Data were retrospectively extracted from electronic medical charts by RBL and JRK. Chart review was initiated in April 2019 and completed for all patients by October 2019. Afterwards, children with no CPB during surgery or major noncardiac surgery were excluded. Children requiring pre‐ or postoperative ECMO were not included in the analysis.

During the study period, TEG became mandatory prior to the delivery of cryoprecipitate. Consequently, children without TEG measurements were excluded from the final analysis. Last, children with missing data on cryoprecipitate transfusion were excluded from the final analysis.

Data collection comprised: Age, gender, diagnosis, and RACHS‐1 category upon admission. Intraoperative data reflect the timespan from induction of anesthesia until the transfer to the cardiac intensive care unit.

Intraoperative data were: CPB‐duration, aortic cross‐clamp time, selective cerebral perfusion time, lowest core temperature, and intraoperative bleeding volume.

Transfusion data included the volume of red blood cells, cryoprecipitate, and platelet concentrate both intra‐ and postoperatively. Transfusion of cryoprecipitate was divided into low and high volume, reflecting below or above the median value.

Hemostatic data were collected in the form of TEG6s and multiplate measurements both at the end of cardiopulmonary bypass and later at the discretion of the treating clinician. However, due to the inconsistent time points for additional TEG6s measurements, only the test performed at the end of CPB was included in the present study.

Bleeding complications were evaluated by measuring intraoperative bleeding volume, including suction pump, weighed gauzes, and loss in surgical drapes. Postoperative bleeding was measured by chest tube output until POD1 at 6:00 AM and whether the child required reoperation before the next morning.

### 2.3 Ethical approval

The study was conducted as a part of a local quality assurance project, and data were collected after institutional approval (case no. 19008302). Due to the retrospective study design, the need for written informed consent was waived.

### 2.4 Statistical analysis

The normality of data distribution was evaluated using visual inspection of Q–Q plots. Data not following a normal distribution are presented as the median and interquartile range (IQR). Categorical variables are expressed as counts and percentages. Comparison of groups was performed using Kruskal–Wallis test for continuous variables, and Pearson's chi‐squared test or Fisher's exact test for categorical variables. A *p*‐value <.05 was considered statistically significant. Effect size calculated as the mean difference with 95% confidence intervals (CI) were applied where relevant.

A sensitivity and specificity analysis, including a receiver operating characteristics (ROC) analysis, was performed to evaluate the ability of the TEG‐FF‐MA measurements to identify patients in need of cryoprecipitate transfusion, and to determine the optimal threshold to predict an intraoperative bleeding volume ≥ 10 ml/kg. A sample size calculation for the area under the ROC curve was performed with a significance level of 5% and a power of 80% and found that at least 62 positive cases and 31 negative controls were needed, assuming a control: case ratio of approximately 0.5.

All statistical analyses were performed with the use of RStudio® software, version 1.2.1335 (RStudio Team [2020]. RStudio: Integrated Development for R. RStudio, PBC, Boston, MA).

## 3 RESULTS

A total of 279 infants underwent cardiac surgery with CPB. Children without TEG measurements (*n* = 96) and nine infants with missing data on cryoprecipitate transfusion were excluded, leaving 174 patients with complete data for analysis (Figure SS1). Among these, 147 (84%) children received cryoprecipitate intraoperatively. The use of cryoprecipitate in the different RACHS‐1 categories are displayed in Table 1. The median cryoprecipitate volume among children receiving cryoprecipitate was 13.5 (8.6–19.6) ml/kg. As shown in Table 1, children receiving cryoprecipitate were younger and administration was more frequent in higher RACHS‐1 categories. TEG‐FF‐MA values were significantly lower in children requiring cryoprecipitate and intraoperative bleeding volume was >4 times higher (Table 1). Postoperative bleeding was not significantly different between patients receiving cryoprecipitate or not.

**TABLE 1:** Demographics, surgical complexity, and perioperative outcome 1

	Total population *n* = 174	+ Cryo *n* = 147	− Cryo *n* = 27	*p*‐Value
Age (days)	71 (10–151)	66 (10–132)	98 (45–204)	*p* = .044
Median (IQR) [range]	[1–356]	[1–356]	[6–309]	
Male *n* (%)	107 (61.5%)	93 (63.3%)	14 (51.9%)	*p* = .263
Hypothermia *n* (%)	21	20 (13.6%)	1 (3.7%)	*p* = .131
RACHS‐1 *n* (%)				
1	2	2 (1.4%)	0 (0%)	*p* < .001
2	75	56 (38.1%)	19 (70.4%)	
3	60	55 (37.4%)	5 (18.5%)	
4	32	30 (20.4%)	2 (7.4%)	
5	2	1 (0.7%)	1 (3.7%)	
6	3	3 (2.0%)	0 (0%)	
Perioperative outcomes				
First IntraOP				
TEG‐FF‐MA, mm	7.8 (5.5–11.4)	7.6 (5.3–11.0)	10.5 (7.3–13.4)	*p* = .004
Median (IQR) [range]	[2.1–22.8]	[2.1–22.8]	[2.9–18.2]	−2.4 (CI: −4.1 to −0.7)
IntraOP bleeding (ml/kg)^a^	18 (9–45)	21 (11–47)	5 (3–13)	*p* < .001
Median (IQR) [range]	[0–358]	[1–358]	[0–100]	29 (CI: 8–50)
PostOP bleeding (ml/h/kg)	1.0 (0.7–1.7)	1.1 (0.7–1.7)	0.9 (0.6–1.7)	*p* = .382
Median (IQR) [range]	[0.2–7.5]	[0.2–7.5]	[0.4–5.7]	0.2 (CI: −0.3 to 0.7)

*Note*: Data are shown as median, interquartile range (IQR), and range or frequency and percentage (%). Relevant mean difference with 95% confidence interval (CI) in the perioperative outcomes is shown adjacent to the *p*‐value.

Abbreviations: Cryo: cryoprecipitate; IntraOP, intraoperative; PostOP, postoperative; RACHS‐1, Risk Adjusted Classification for Congenital Heart Surgery 1; TEG‐FF‐MA: thromboelastography functional fibrinogen maximal amplitude.

^a^ +Cryo *n* = 127, −Cryo *n* = 25, total population *n* = 152.

**FIGURE 1:**
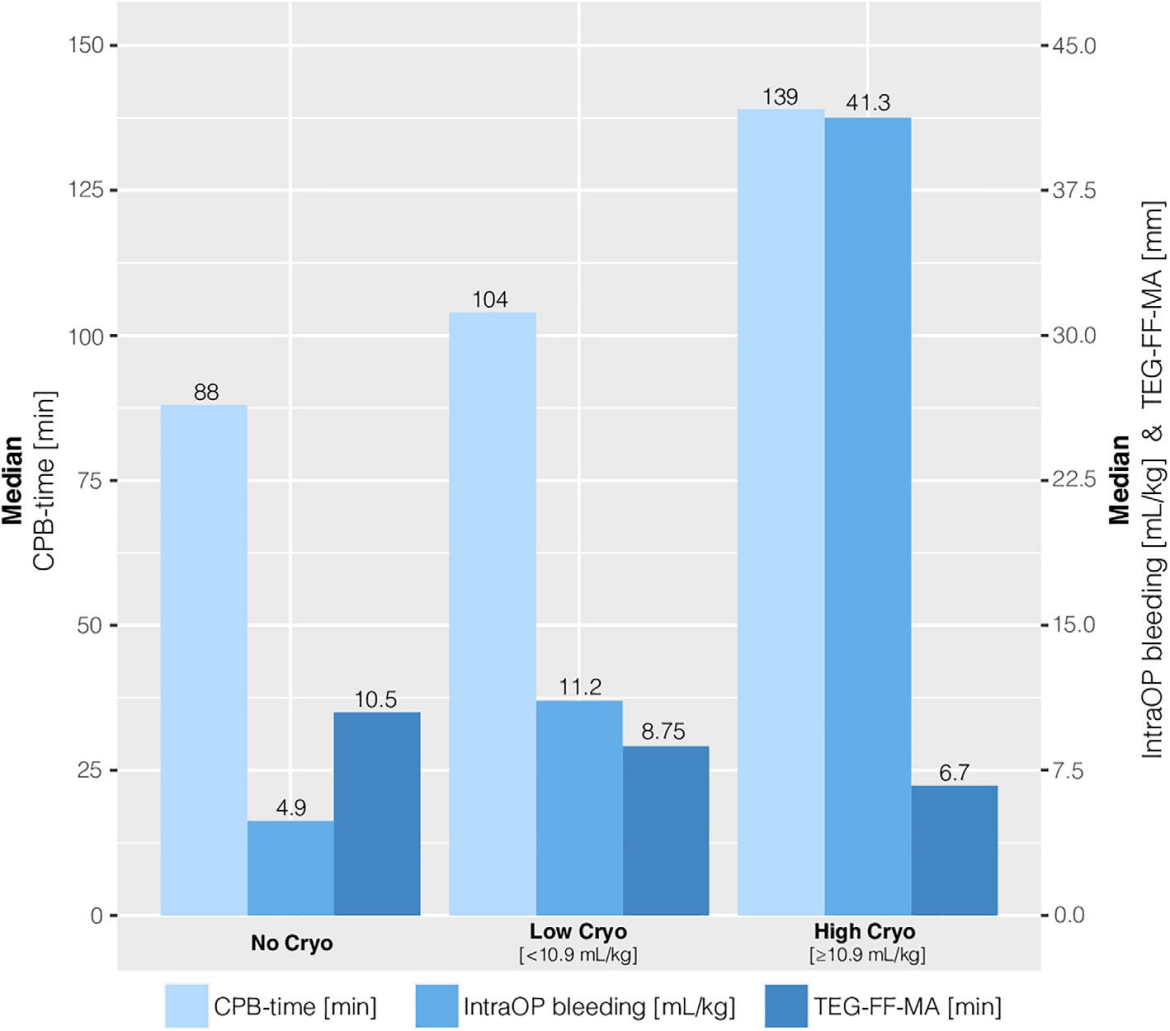
CPB‐time, intraoperative bleeding, and TEG‐FF‐MA measurements in relation to cryoprecipitate substitution. IntraOP, intraoperative; CPB, cardiopulmonary bypass; Cryo, cryoprecipitate; TEG‐FF‐MA, thromboelastography functional fibrinogen maximal amplitude.

**FIGURE 2:**
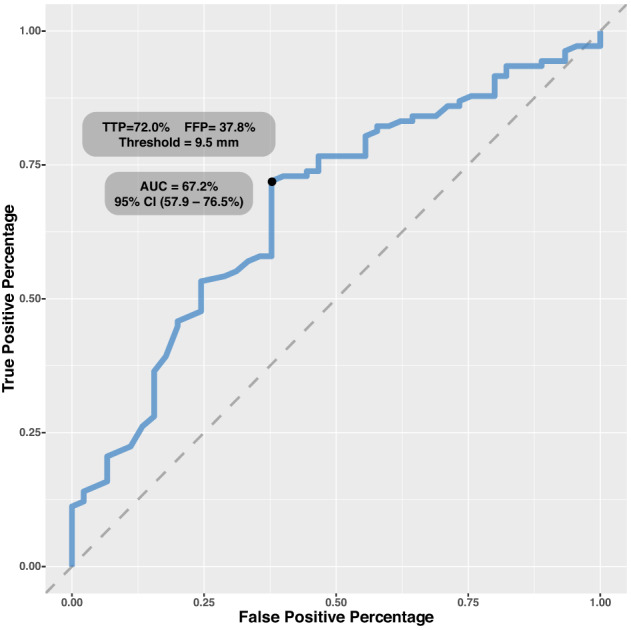
ROC‐analysis for TEG‐FF‐MA and intraoperative bleeding. High‐bleeders are defined as patients with an intraoperative bleeding of ≥10 ml/kg, while Low‐bleeders are patients with <10 ml/kg intraoperative bleeding. AUC, area under curve; FPP, false positive percentage; TTP, true positive percentage.

Cryoprecipitate administration was divided into low or high volume (>10.9 ml/kg), and the amount given depended on CPB‐duration, TEG‐FF‐MA values immediately after CPB, and in particular, intraoperative blood loss (Figure 1). A TEG‐FF‐MA below 10.0 mm is considered the clinical threshold for cryoprecipitate substitution in a bleeding child. ROC curve analysis for the present cohort based on 107 positive cases and 45 negative controls revealed a comparable threshold of 9.5 mm, but with an AUC of only 67% (95% CI: 0.58–0.77) (Figure 2). A threshold of TEG‐FF‐MA < 10.0 mm resulted in the present study in a sensitivity of 74% and a specificity of 56%, which leads to a positive predictive value of 80%, but a negative predictive value of only 47% (Table 2).

**TABLE 2:** Sensitivity and specificity analysis

IntraOP bleeding	TEG‐FF‐MA		
	<10 mm	≥10 mm	
<10 ml/kg	20	25	Specificity: 55.6%
≥10 ml/kg	79	28	Sensitivity: 73.8%
	PPV: 79.8%	NPV: 47.2%	

*Note*: *n* = 152, as 22 patients had missing data for intraoperative bleeding volumes. Data are shown as absolute numbers of patients.

Abbreviations: IntraOP, intraoperative; NPV, negative predictive value; PPV, positive predictive value; TEG‐FF‐MA, thromboelastography functional fibrinogen maximal amplitude.

## 4 DISCUSSION

Eighty‐four percent of the children were transfused with cryoprecipitate. Children receiving cryoprecipitate were younger, underwent more complex surgery and suffered from greater intraoperative blood losses. Almost all children having surgery requiring hypothermia received cryoprecipitate. The administered volume of cryoprecipitate was associated with CPB‐time and intraoperative blood loss as well as TEG‐FF‐MA values. A TEG‐FF‐MA value <10 mm identified almost 4 out of 5 children requiring fibrinogen substitution, but at the same time, the negative predictive value was less than 50%.

Similar patient characteristics have previously been associated with cryoprecipitate transfusion, including low age and longer CPB duration.^2^, ^3^, ^4^, ^11^ Due to the retrospective study design, we cannot conclude whether young age on its own is an independent risk factor for transfusion, or simply associated with higher RACHS‐1 categories. Still, the present observations confirm previous studies identifying young age and higher RACHS‐1 categories as independent risk factors for bleeding and transfusion.^12^, ^13^ A high RACHS‐1 category is associated with more complex surgery and consequently extended CPB‐time. This close interaction between age, surgical complexity, and duration of CPB makes it difficult to evaluate the contribution from individual components in the risk calculation.

As a standard procedure, TEG6s was taken routinely when weaning from CPB, and in accordance with international guidelines, transfusion of blood products in the presence of bleeding was guided by the TEG6s results. This practice is based on numerous observational studies from recent years, reporting that results from point‐of‐care tests like TEG6s and ROTEM are both closely correlated with actual fibrinogen concentrations, and that post‐CPB point‐of‐care tests are effective in predicting both intraoperative and postoperative excessive bleeding in children undergoing congenital heart surgery.^14^, ^15^, ^16^ However, pediatric studies prospectively evaluating point‐of‐care tests for transfusion guidance are sparse and highly warranted.^17^

The volume of cryoprecipitate transfused was associated with TEG‐FF‐MA level, but also CPB time, and intraoperative bleeding volume. Again, the complex interaction between these risk factors makes it difficult to isolate the contribution from individual components. As seen from a positive predictive value of 80%, having a low fibrinogen level at the time of weaning off bypass increases the risk of developing coagulopathy during surgical hemostasis. On the other hand, with a negative predictive value of 47%, a normal TEG‐FF‐MA coming off bypass does not prevent the development of impaired hemostasis due to consumption coagulopathy, which is especially the case in complex surgery with prolonged CPB duration, extensive vascular anastomosis, and increased intraoperative bleeding. Unfortunately, due to the retrospective study design, we cannot deduce the contribution of additional TEG6s measurements, since additional samples were taken dependent on a clinical assessment and not in a systematic manner. However, we suggest, that rather than relying on a single‐point TEG measurement, additional measurements may be useful as guidance of when to transfuse after initial hemostasis. This concept is further supported by Ranucci et al., who demonstrated a significant association between fibrinogen levels at the time of ICU arrival and postoperative chest drain losses, but not between fibrinogen levels after CPB and chest drain production.^11^

When comparing observations from the present study with reports from adult cardiac surgery, a similar picture is emerging. A close correlation has been reported between point‐of‐care test results and actual fibrinogen concentration in adults, especially before hemostatic therapy.^18^, ^19^

In addition, a recent meta‐analysis reported that point‐of‐care guided transfusion reduces allogenic blood product transfusion, postoperative bleeding, and need for redo surgery.^20^

It must be emphasized, that even though major effort has been devoted to finding an optimal threshold in point‐of‐care tests for fibrinogen administration, and several thresholds have been published, a clear cut‐off level has yet to be identified. Due to these uncertainties, unnecessary treatment may occur when utilizing point‐of‐care test‐guided fibrinogen substitution, thereby increasing the risk of thrombosis.^9^, ^21^, ^22^

The difficulties in identifying the optimal fibrinogen threshold on point‐of‐care tests may relate to postoperative fibrinogen kinetics. In this setting, its role as a positive acute phase reactant may lead to fast recovery of fibrinogen levels. As long as the increase in fibrinogen concentration is not outdone by massive bleeding, fibrinogen levels will recover spontaneously within a few hours after surgery.^23^ Furthermore, evidence is emerging that point‐of‐care tests do not accurately reflect fibrinogen concentrations, which may explain some of the conflicting results previously reported.^24^ Finally, recent transfusion audits have estimated that up to 24% of cryoprecipitate is transfused inappropriately in relation to current transfusion guidelines.^25^, ^26^

Unbalanced hemostasis/thrombosis may be a possible consequence of unnecessary treatment with fibrinogen, and while no thrombotic complications were observed in this study population, a previous study has identified thrombotic complications as a possible consequence of over‐treatment. This, combined with the known risks of increased donor exposure and transfusion risks in general, underlines the need for a balanced approach.^27^

In summary, while the use of point‐of‐care test‐guided hemostasis is the cornerstone in pediatric cardiac surgery, findings of the present study indicate that interpretation of TEG6s is valuable, but not the only determinant of substitution. Transfusion requirements also rely on clinical experience, which became evident when we compared cryoprecipitate transfusion in a comparable, but smaller group of patients, where TEG6s measurements were not available (see Table SS1). In addition, while 84% of infants with TEG6s measurements received cryoprecipitate, the distribution of cryoprecipitate in patients without TEG6s measurements was almost identical to the present study population. These observations indicate, that in a group of infants undergoing complex surgery with higher RACHS‐1 classification (Table 1), the contribution from TEG6s has limited influence on the amount of cryoprecipitate transfused, instead a close relationship was seen with the bleeding risk of the surgical procedure itself. This notion is further supported by the fact that the presence of hypofibrinogenemia did not require transfusion of cryoprecipitate in the absence of bleeding. Among infants not receiving cryoprecipitate, almost 50% had a TEG‐FF‐MA level below or equal to 10 mm. Despite a low TEG‐FF‐MA level, these children had an intraoperative blood loss of less than 10 ml/kg (data not shown). All in all, these findings indicate that while abnormal TEG‐FF‐MA values indicate a need of cryoprecipitate transfusion, this is not the case in every circumstance, especially in the absence of bleeding, since other factors apart from TEG‐values seem to affect the need for cryoprecipitate transfusion. Future prospective studies are needed to further examine this relationship.

Limitations of the present study include the fact that it is a single‐center study, which may limit the generalizability to other centers. Second, the variation in the surgical procedures, surgeons, anesthetists, and the combination thereof may influence the outcome, especially due to the retrospective nature of the study design. Last, no clear‐cut normal ranges for TEG6s have been established for pediatric cardiac patients in earlier studies. Indeed, despite the fact that experienced pediatric anesthesiologists, in a recent review of studies evaluating the benefit of viscoelastic tests in pediatric surgery, emphasize that fibrinogen has a major role in preventing postoperative bleeding, the applied reference level for fibrinogen substitution is 7 and 10 mm for the similar FIBTEM test in their respective pediatric centers.^17^ The underlying argument for choosing different thresholds is not clear, but often each center develops their own cut‐off level. However, due to the observational design of the present study, we cannot claim that the chosen cut‐off value of TEG‐FF‐MA at 10 mm was better or worse than any other value. Nevertheless, the ROC‐analysis indicated an optimal cut‐off of 9.5 mm, close to the clinical standard.

On the other hand, a strength of the present study was the changing of institutional guidelines during data collection. TEG measurement became mandatory prior to the delivery of cryoprecipitate, which enabled us to compare children transfused with cryoprecipitate based on TEG‐FF‐MA results and children managed without. It is also a strength that the children's age was restricted to include only infants, making the group more homogenous, although developmental hemostasis may influence the outcome. Unfortunately, it was not possible to separate the impact of age by looking at, for instance, neonates exclusively, since the surgical procedure is closely correlated to age, too.

In conclusion, the study identified several risk factors for cryoprecipitate administration and the transfused volume. In addition, this study is the first to present data on thresholds and predictive values for the use of TEG6s in guiding cryoprecipitate transfusion in pediatric cardiac surgery. Perioperative factors apart from TEG6s measurements have a major influence on cryoprecipitate transfusion. Observations from earlier studies indicate that TEG6s measurements may be more informative at a later stage when initial surgical hemostasis has been achieved. However, future studies are needed to evaluate if serial measurements can improve the prognostic ability of TEG6S and prevent bleeding. Finally, follow‐up studies focusing on thrombotic complications should be performed to ensure a balanced approach between hemostasis and thrombosis.

## AUTHOR CONTRIBUTIONS

All authors participated in the study design, revised the manuscript, and approved the final version. In addition to this: R.B.L.: Data collection, analysis, interpretation and drafting the manuscript. J.R.K.: Data collection. H.B.R.: Analysis, interpretation, and drafting the manuscript.

## CONFLICT OF INTEREST

The authors declare that they have no conflict of interest.

## Supporting information


**Figure S1** STROBE chart
CPB: cardiopulmonary bypass, ECMO: extracorporeal membrane oxygenation, TEG: thromboelastography.

**Table S1** Demographics in relation to whether TEG monitoring was applied.
Data are shown as median and interquartile range (IQR) or frequency and percentage (%). TEG‐FF‐MA: Thromboelastography functional fibrinogen maximal amplitude, Cryo: cryoprecipitate, RACHS‐1: Risk Adjusted Classification for Congenital Heart Surgery 1, IntraOP: intraoperative, PostOP: postoperative.
†: +TEG/+Cryo *n* = 127, +TEG/‐Cryo *n* = 25, −TEG/+Cryo *n* = 36, −TEG/‐Cryo *n* = 42
