# Supplementary figures and images for: Evaluation of Thromboelastography 6s prognostication of fibrinogen supplementation in pediatric cardiac surgery
Source: Acta Anaesthesiol Scand. 2022 Sep 11;66(10):1166–73. doi: 10.1111/aas.14144 (PMC9826011; doi:10.1111/aas.14144)

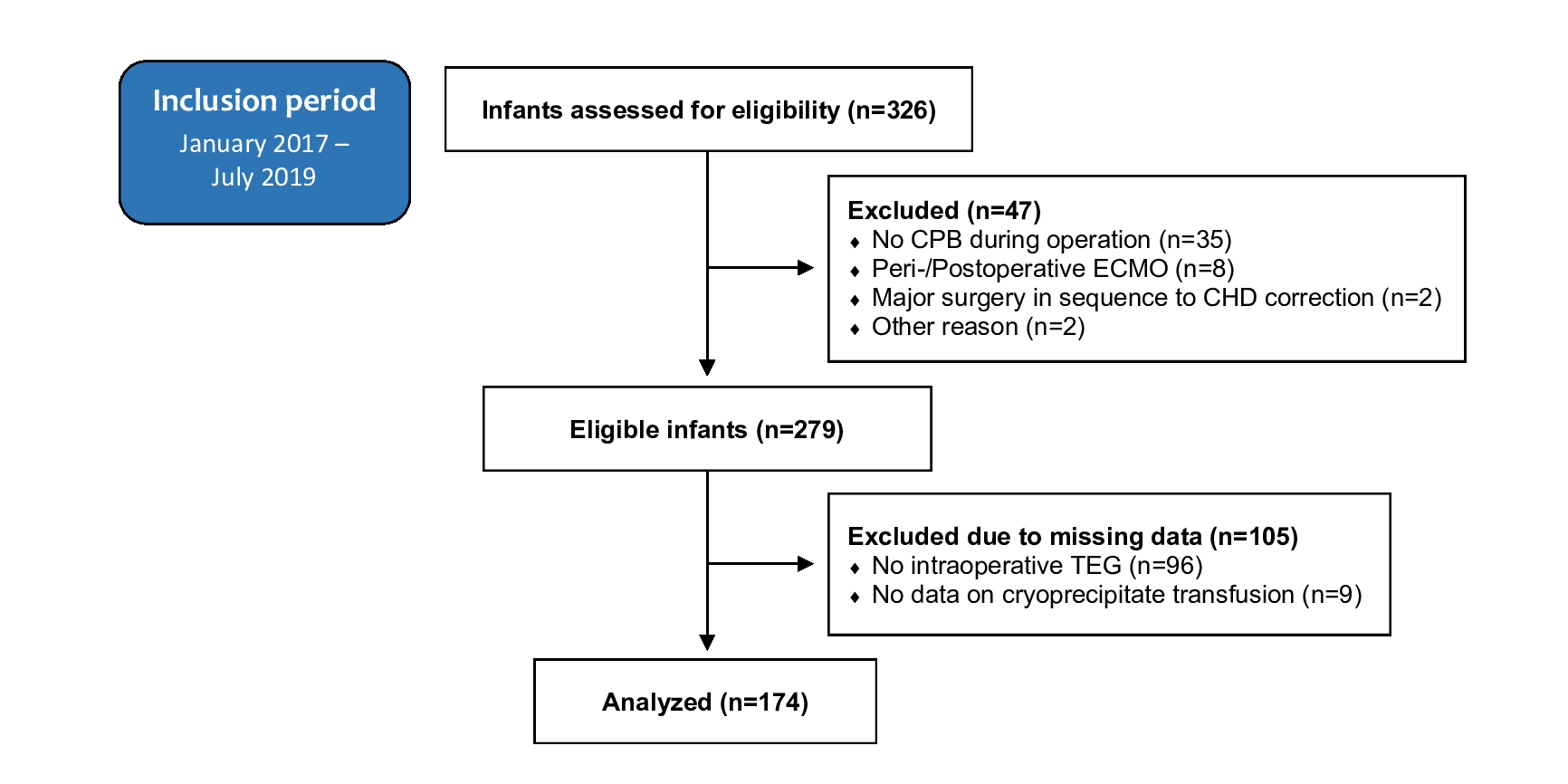

Supplement: Supplementary file 1 — Figure S1 STROBE chart CPB: cardiopulmonary bypass, ECMO: extracorporeal membrane oxygenation, TEG: thromboelastography. [file AAS-66-1166-s001.jpeg]
