# Supplementary material for: Evaluation of Thromboelastography 6s prognostication of fibrinogen supplementation in pediatric cardiac surgery
Source: Acta Anaesthesiol Scand. 2022 Sep 11;66(10):1166–73. doi: 10.1111/aas.14144 (PMC9826011; doi:10.1111/aas.14144)
Supplement: Supplementary file 2 — Table S1 Demographics in relation to whether TEG monitoring was applied. Data are shown as median and interquartile range (IQR) or frequency and percentage (%). TEG‐FF‐MA: Thromboelastography functional fibrinogen maximal amplitude, Cryo: cryoprecipitate, RACHS‐1: Risk Adjusted Classification for Congenital Heart Surgery 1, IntraOP: intraoperative, PostOP: postoperative. †: +TEG/+Cryo n = 127, +TEG/‐Cryo n = 25, −TEG/+Cryo n = 36, −TEG/‐Cryo n = 42 [file AAS-66-1166-s002.docx]

|  | | **+ TEG** | | **-TEG** | |
| --- | --- | --- | --- | --- | --- |
|  |  | **+ Cryo**  n = 147 | **- Cryo**  n = 27 | **+ Cryo**  n = 41 | **-Cryo**  n = 55 |
| Age, days  Median (IQR) [Range] | | 66 (10 – 132) [1 – 356] | 98 (45 – 204)  [6 – 309] | 84 (10 – 162)  [4 – 283] | 134 (67 – 193)  [6 – 350] |
| Male n (%) | | 93 (63.3%) | 14 (51.9%) | 21 (51.2%) | 32 (58.2%) |
| Hypothermia  n (%) | | 20 (13.6%) | 1 (3.7%) | 8 (19.5%) | 1 (1.8%) |
| RACHS-1  n (%) | 1 | 2 (1.4%) | 0 (0%) | 0 (0%) | 5 (9.1%) |
|  | 2 | 56 (38.1%) | 19 (70.4%) | 16 (39.0%) | 39 (70.9%) |
|  | 3 | 55 (37.4%) | 5 (18.5%) | 14 (34.1%) | 8 (14.5%) |
|  | 4 | 30 (20.4%) | 2 (7.4%) | 9 (22.0%) | 2 (3.6%) |
|  | 5 | 1 (0.7%) | 1 (3.7%) | 0 (0%) | 0 (0%) |
|  | 6 | 3 (2.0%) | 0 (0%) | 3 (7.3%) | 0 (0%) |
| **Perioperative outcomes** | | | | | |
| First IntraOP  TEG-FF-MA, mm Median (IQR) [Range] | | 7.6 (5.3 – 11.0)  [2.1 – 22.8] | 10.5 (7.3 – 13.4)  [2.9 – 18.2] |  |  |
| IntraOP Bleeding, mL/kg^✝^  Median (IQR) [Range] | | 21 (11 – 47)  [1 – 358] | 5 (3 – 13)  [0 – 100] | 12 (7 – 44) [2 – 241] | 6 (4 – 13)  [1 – 39] |
| PostOP Bleeding, mL/hour/kg Median (IQR) [Range] | | 1.1 (0.7 – 1.7) [0.2 – 7.5] | 0.9 (0.6 – 1.7)  [0.4 – 5.7] | 1.3 (0.9 – 1.5)  [0.2 – 4.3] | 1.0 (0.8 – 1.4)  [0.0 – 2.4] |

**Supplementary Table 1:** Demographics in relation to whether TEG monitoring was applied.
Data are shown as median and interquartile range (IQR) or frequency and percentage (%). TEG-FF-MA: Thromboelastography functional fibrinogen maximal amplitude, Cryo: cryoprecipitate, RACHS-1: Risk Adjusted Classification for Congenital Heart Surgery 1, IntraOP: intraoperative, PostOP: postoperative.
✝: +TEG/+Cryo n = 127, +TEG/-Cryo n = 25, -TEG/+Cryo n = 36, -TEG/-Cryo n = 42
